# Characterization and genomic analysis of a novel halovirus infecting *Chromohalobacter beijerinckii*

**DOI:** 10.3389/fmicb.2022.1041471

**Published:** 2022-12-07

**Authors:** Hao Yi, Chaoqun Fu, Kaixin Diao, Zhiying Li, Xiaolong Cui, Wei Xiao

**Affiliations:** Yunnan Institute of Microbiology, College of Life Science, Yunnan University, Kunming, China

**Keywords:** *Chromohalobacter*, bacteriophage, genome, non-heme iron enzymes, hypersaline environments

## Abstract

Bacteriophages function as a regulator of host communities and metabolism. Many phages have been isolated and sequenced in environments such as the ocean, but very little is known about hypersaline environments. Phages infecting members of the genus *Chromohalobacter* remain poorly understood, and no *Chromohalobacter* phage genome has been reported. In this study, a halovirus infecting *Chromohalobacter* sp. F3, YPCBV-1, was isolated from Yipinglang salt mine. YPCBV-1 could only infect host strain F3 with burst size of 6.3 PFU/cell. It could produce progeny in 5%–20% (w/v) NaCl with an optimal concentration of 10% (w/v), but the optimal adsorption NaCl concentration was 5%–8% (w/v). YPCBV-1 is sensitive to pure water and depends on NaCl or KCl solutions to survive. YPCBV-1 stability increased with increasing salinity but decreased in NaCl saturated solutions, and it has a broader salinity adaptation than the host. YPCBV-1 has a double-stranded DNA of 36,002 bp with a G + C content of 67.09% and contains a total of 55 predicted ORFs and no tRNA genes. Phylogenetic analysis and genomic network analysis suggested that YPCBV-1 is a novel Mu-like phage under the class Caudoviricetes. Auxiliary metabolic gene, SUMF1/EgtB/PvdO family non-heme iron enzyme, with possible roles in antioxidant was found in YPCBV-1. Moreover, DGR-associated genes were predicted in YPCBV-1 genome, which potentially produce hypervariable phage tail fiber. These findings shed light on the halovirus-host interaction in hypersaline environments.

## Introduction

Viruses are noncellular organisms and exist wherever life is found (Suttle, 2005). They are the most abundant biological entity on earth, with an estimated population of 10^31^ (Cobián-Güemes et al., 2016), an order of magnitude greater than their hosts (Wommack and Colwell, 2000; Suttle, 2007). Viruses play an important role in processes such as material cycling, energy flow, and host community structure regulation (Suttle, 2005; Suttle, 2007). Viruses inhabiting hypersaline environments, such as salt mines and salt lakes, are called haloviruses (Oren, 2002; Atanasova et al., 2016), and the number of viruses in some hypersaline waters can be as high as 10^10^ VLP/ml (Boujelben et al., 2012).

Haloarchaea are dominant in hypersaline environments, where halobacteria may account for 20% of the microbiota (Oren et al., 1997; Ghai et al., 2011). The majority of haloviruses isolated to date are haloarchaeal viruses, with a few halobacterial viruses. Since the isolation of the first halobacterial virus F9-11 in a hypersaline soil in 1988 (Calvo et al., 1988), a total of 29 halobacterial viruses have been reported (Calvo et al., 1988, 1991; Kauri et al., 1991; Calvo et al., 1994; Goel et al., 1996; Seaman and Day, 2007; Mobberley et al., 2008; Kukkaro and Bamford, 2009; Aalto et al., 2012; Atanasova et al., 2012; Shen et al., 2012; Yu et al., 2015; Atanasova et al., 2015b; Fu et al., 2016; Villamor et al., 2017; Wang and Li, 2018; Rodela et al., 2019; Zrelovs et al., 2020; Olonade et al., 2021; Wang et al., 2022), with hosts including *Halomonas*, *Salicola*, *Pseudomonas*, *Salinivibrio*, *Salisaeta*, *Salinibacter*, *Chromohalobacter*, and *Virgibacillus*. Most studies have focused on virus morphology as well as physiological and biochemical characteristics, while only 15 halobacterial virus genomes have been published in GenBank.

*Chromohalobacter* is a moderate halobacteria commonly found in hypersaline environments (Zhang et al., 2021; Lülf et al., 2022; Sirichoat et al., 2022), and some strains of *Chromohalobacter* are considered to have potential value in biotechnology due to their ability to produce tetrahydropyrimidines, proteases, and lipases (Vidyasagar et al., 2006; Rodríguez-Moya et al., 2013; Salar-García et al., 2017; Ai et al., 2018; Tanaka et al., 2020). To the best of our knowledge, only one phage infecting *Chromohalobacter*, JMT-1, has been isolated, from the saline lake Yuncheng, China, using *Chromohalobacter* sp. LY7-3 as a host (Wang and Li, 2018), but its genome has not been sequenced. In this study, the halovirus YPCBV-1 was isolated from Yipinglang salt mine in Yunnan Province using *C. beijerinckii* F3 as a host. It was found to possess SUMF1/EgtB/PvdO family non-heme iron enzyme and represented a novel phage. Combined with the biological features, this study provides new insights into the possible interactions between hosts and halovirus.

## Materials and methods

### Isolation of host *Chromohalobacter* sp. F3 and phage YPCBV-1

*Chromohalobacter* sp. F3 and its phage YPCBV-1 were both isolated from saline soil samples collected from the Yipinglang salt mine in Yunnan Province (101°90′E, 25°28′N), China (Xiao et al., 2013). The host was isolated by the standard dilution-plating technique on Marine agar 2,216 (MA, Difco), while the strains were purified and cultured in modified LB (MLB, NaCl 100 g/l, tryptone 8.0 g/l, yeast extract 4.0 g/l, [pH 7.2–7.6]).

To obtain the phages enrichment, 23 g of soil samples and 1 ml host culture were inoculated into 100 ml of MLB liquid medium and shaken at 28°C, 120 rpm for 48 h, centrifuged at 6000 × g for 15 min, the supernatant was collected and filtered through 0.22 μm pore-size filter (Millipore). Subsequently, the filtered lysate was diluted and mixed with 0.5 ml exponentially growing host culture. After adsorption for 15 min at 28°C, the mixture was added to 4 ml of the semi-solid medium, which was then poured onto a pre-prepared solid monolayer plate. The double-layer plates were incubated right-side-up at 28°C and examined for the presence of plaques. Individual plaques were selected and purified three times by repeated single-plaque propagation on strain F3 (Fu et al., 2016).

### Transmission electron microscopy

To determine the morphologies of the host cells and phage virions, the cell or phage suspensions were stained with 2% (w/v) sodium phosphotungstate for 1 min, air dried, and placed under TEM (JEM-2100; 200 kV) to observe the morphology.

### Phylogenetic analysis

16S rRNA gene of strains were compared at the Ezbiocloud.^1^ Phylogenetic trees were constructed from similarity sequences using the neighbor-joining method by Mega 7.0 (Kumar et al., 2016). All parameters are default except the Bootstrap value is 1,000, the p-distance model is used to calculate the distance, and the Gap/Missing Data Treatment cutoff of 50%. Phylogenetic analysis was carried out based on the amino acid sequences of the gene encoding major capsid proteins (MCP) of the YPCBV-1 and 20 best hit viruses found in the GenBank database, using a BLASTp with E-value cutoff of 1e-05. The phylogenetic tree including YPCBV-1 genome and 20 best hit genomes in GenBank was generated using the VICTOR (Meier-Kolthoff and Goker, 2017) online server^2^ with default parameters. In addition, 15 halobacterial virus genomes and best hit viruses in network analysis were including in the genome tree.

### Salinity range and optimal salinity for host growth

To test NaCl tolerance, strain F3 were cultured in liquid MLB with different concentrations of NaCl (0, 2, 5, 8, 10, 12, 15, 20, and 25% [w/v]) at 28°C, 160 rpm for 10 h and the OD600 was determined. The experiment was conducted in single replicate.

### Host range

The host range was assessed using other 7 bacteria: three halobacteria isolated from the Yipinglang salt mine (*Chromohalobacter canadensis* F7、*Halomonas titanicae* H5) and Qiaohou salt mine (*H. ventosae* QH52-2)in Yunnan by our laboratory (Fu et al., 2016), four *Chromohalobacter* strains purchased from the China General Microbiological Culture Collection Center (CGMCC; *C. beijerinckii* 1.9020、*C. japonicus* 1.7474、*C. canadensis* 1.7979、*C. marismotui* 1.2321). Virus suspensions were dropped on MLB solid medium spread with potential hosts and incubated at 28°C for 24 h to observe plaque formation.

### One-step growth curve

The host was cultured with MLB liquid medium to OD600 = 0.3 and then centrifuged at 5,000 × g for 10 min. The phage suspension (10^8^–10^9^ PFU/ml) was mixed with host at MOI = 1. After adsorbed for 15 min at 28°C. The cells were collected by centrifugation at 10,000 × g for 10 min and resuspended in 1 ml of fresh medium. This process was repeated 3 times to remove unadsorbed phage particles. The cells were added to liquid medium (50 ml) and incubated at 28°C, 120 rpm for 120 min. The titers were determined by double-layer plates at the indicative time. The experiment was conducted in triplicate.

### Sensitivity to pH, temperature, organic solvent, detergents, and proteinase K

The phage suspensions (10^8^–10^9^ PFU/ml) were incubated at 28, 50, 60, and 70°C and sampled at intervals. In addition, phage suspensions were inoculated in MLB liquid medium with different pH (3–12) at 28°C for 1 h. Phage suspensions and anhydrous ethanol were mixed at a volume ratio of 1:9 and incubated at 28°C for 30 min. Chloroform was added to the phage suspension to the final concentration of 20% and incubated at 120 rpm, 28°C for 12 h. Proteinase K was added to the phage suspension to the final concentration of 2 mg/ml, incubated at 56°C for 1 h. SDS solution was added to the phage suspensions to the final concentration of 0.1% (w/v) and incubated at 28°C for 1 h. Phage titer were tested by the double-layer agar plate method at the indicative time. Except for temperature, other experiments were conducted in triplicate.

Percent survival = (phage titer detected after experiment / initial phage titer) × 100%.

### Effect of salinity and ions on phage stability

To assess the viability of free phage particles in a hypersaline environment, we analyzed the stability of YPCBV-1 over 28 days at different salt concentrations. An aliquot of 10 μl of phage suspensions (10^8^–10^9^ PFU/ml) was inoculated in 0, 1, 5, 10, 15, 20, 25, and 30% (w/v) NaCl or KCl solutions. Similarly, phage suspensions were inoculated in NaBr or CaCl2 solutions (0, 1, 10, 20, and 30% [w/v]). The phage titers were determined by double-layer agar plate method at the indicative time. The percent survival were calculated as described above. The experiment was conducted in triplicate.

### Salinity range of phage infecting host

Phage infection assays (MOI = 1) were conducted in liquid MLB with different NaCl concentrations of 0%, 2%, 5%, 8%, 10%, 12%, 15%, 20%, 25%, and 30% (w/v). The phage titers were tested by double-layer agar plate method at the end of the exponential phase. The experiment was conducted in single replicate.

### Effect of salinity on phage adsorption

The log phase host strain F3 and phage lysate were mixed (MOI = 1) in liquid MLB with different NaCl concentrations of 0%, 5%, 8%, 10%, 12%, 15%, 20%, 25%, and 30% (w/v). Adsorption for 15 min at room temperature, centrifugation at 10,000 × g for 5 min, phage amounts in supernatant were test by double-layer agar plate method. The experiment was conducted in triplicate. The percent of adsorption at 10% salinity was considered as 100%.

Percent adsorption = (initial phage titer − phage titer in supernatant)/ initial phage titer × 100%.

### Extraction and restriction endonuclease digestion of phage DNA

Enrichment and concentration of phage particles were carried out as described previously (John et al., 2011; Fu et al., 2016). Briefly, the purified phage particles (10^9^ PFU/ml) were filtered through 0.22 μm pore size filter, then treated with DNase I and RNase A, and incubated at 37°C for 1 h. The genomic DNA of phages was extracted using the TIANamp Virus DNA/RNA Kit (TIANGEN, China) according to the manufacturer’s instructions. The purified phage DNA was treated with restriction endonucleases *Eco*RI, *Xho*I, *Bam*HI, and *Hind*III, followed by 1% (w/v) agarose gel electrophoresis for detection.

### Genome analysis

To construct the DNA library, the DNA was first fragmented using Covaris M220, followed by end-flattening, addition of A and adapter. The target fragments were selected by agarose gel electrophoresis and then PCR was performed to amplify the target fragments (TruSeq™ DNA Sample Prep Kit). After the PCR product is purified by magnetic particles, the library is qualified by detecting the length (Agilent Bioanalyzer), concentration (Qubit) and A260/280 (Nanodrop). DNA library was sequenced at the Majorbio Cooperation (Shanghai, China) using Illumina MiSeq (PE250). Controls were performed using trimmomatic (Bolger et al., 2014). The adapter sequences in the reads are first removed, and then the bases containing non-A, G, C, and T at the 5′ end are removed before trimming. The reads with low sequencing quality (sequencing quality value less than Q20) were trimmed and the reads containing N up to 10% were removed. Finally, we discard the adapter and the small fragments with a length of less than 25 bp after quality trimming. The optimized sequences were assembled with multiple Kmer parameters using SOAP denovo (v2.04; Luo et al., 2012) to obtain the optimal assembly results. Next, GapCloser (v1.12) was used to perform gap filling and base correction on the assembly results. Then Barrnap (v0.4.2)^3^ and tRNAscan-SE (v1.3.1; Chan and Lowe, 2019) were used to predict the rRNA and tRNA in the genome, respectively. Gene prediction was performed using Prokka (v1.14.6; Seemann, 2014). The predicted gene sequences were Blastx-matched (Altschul et al., 1997) against the GenBank nr database to obtain annotation information. Phage genome mapping was performed using SnapGene Viewer (v6.0.2; from Insightful Science; available at^4^). Conserved structural domains were matched against the Conserved domains Database (CDD; Marchler-Bauer et al., 2002). And whole genome sequences were compared with viral sequences in GenBank by Blast. Similarities between phage whole genomes were calculated by VIRIDIC using default parameters (Moraru et al., 2020). Genomic network analysis was performed by vConTACT2 (v0.9.19; Jang et al., 2019), ClusterONE (v1.0; Nepusz et al., 2012), and Cytoscape (v3.9.1; Shannon et al., 2003). The phage genome was submitted to NCBI with the accession number OP380511.

## Results

### Biological features of phage YPCBV-1

**Figure 1:**
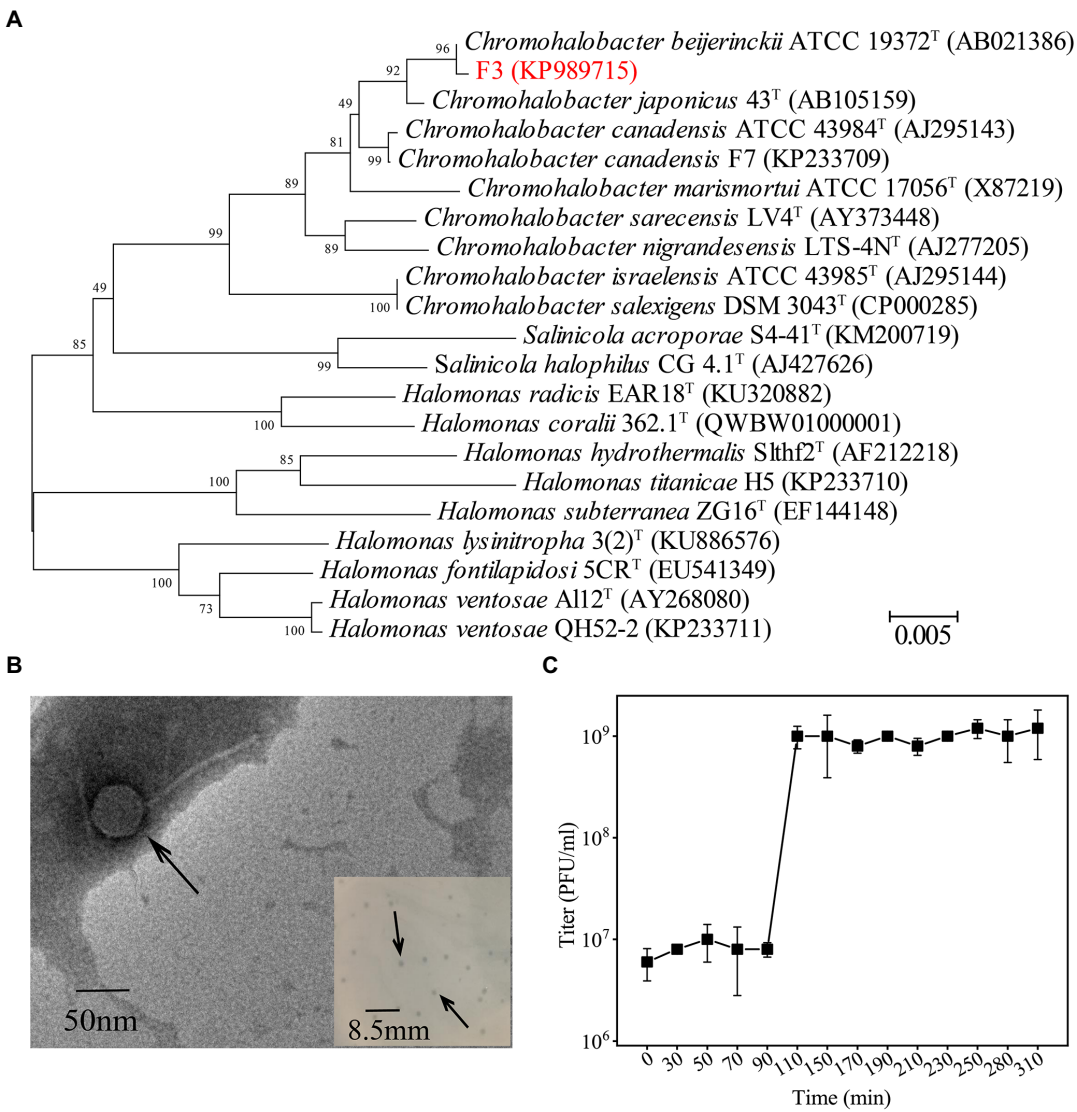
**(A)** Phylogenetic analysis of F3 based on the 16S rRNA gene sequence. Phylogenetic trees were constructed using the neighbor-joining method by Mega 7.0. All parameters are default except the Bootstrap value is 1,000, the p-distance model is used to calculate the distance, and the Gap/Missing Data Treatment cutoff of 50%. **(B)** TEM micrographs of YPCBV-1. Scale bar, 50 nm. Inset shows plaques morphology of YPCBV-1. Scale bar, 8.5 mm. **(C)** One-step growth curve of YPCBV-1. Error bars represent the standard deviation of three replicates.

Strain F3 was isolated from the saline soil collected from Yipinglang salt mine using MBA medium. Phylogenetic analysis based on 16S rRNA gene sequences showed that F3 clustered with *Chromohalobacter. beijerinckii* ATCC 19372^T^, with 98.74% identity (Figure 1A). F3 showed pale yellow opaque round colonies on solid MLB medium. The cell was rod-shaped with a length of 1.2–2.1 μm and a width of 0.46–0.6 μm (Supplementary Figures S1, S2). F3 can grow at NaCl concentrations of 2%–25% (w/v) and has an optimal growth salinity of 8% (w/v; Supplementary Figure S3).

The phage Yipinglang Chromohalobacter Beijerinckii Virus 1 (YPCBV-1) was isolated from the saline soil from Yipinglang salt mine using F3 as the host. YPCBV-1 produced plaques with a diameter of 0.5–1.5 mm after 24 h (Figure 1B). TEM analysis revealed YPCBV-1 exhibits head-tail morphology with a head diameter of approximately 54 ± 8 (*n* = 4) and a tail length of approximately 136 ± 7 (*n* = 4; Figure 1B). The one-step growth curve showed a burst size of 6.3 PFU/cell at 10% (w/v) NaCl concentration (Figure 1C). The host range of YPCBV-1was narrow and only infected the host F3, but not *C. beijerinckii* CGMCC 1.9020. The YPCBV-1 genome could be cleaved by *Eco*R I and *Xho* I but not by *Bam*H I and *Hind* III (Supplementary Figure S4), indicating the genome is double-stranded DNA.

### Response of YPCBV-1 to salinity

**Figure 2:**
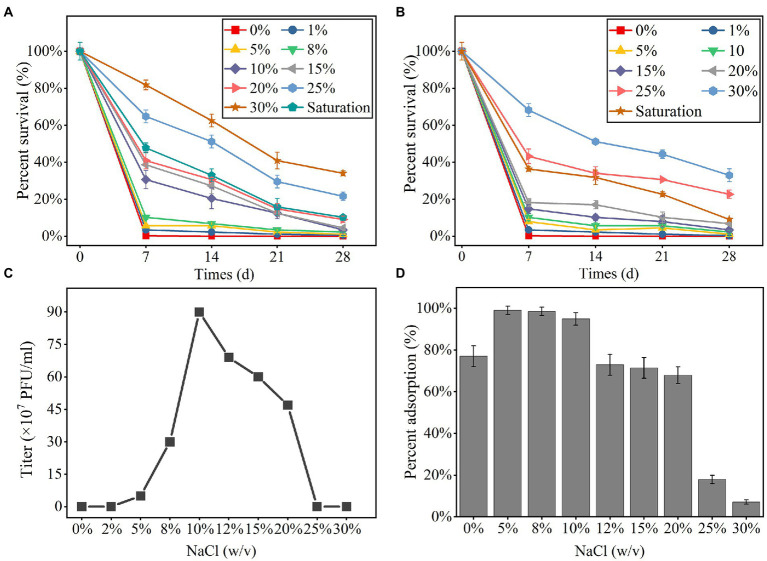
The percent survival of YPCBV-1 at different NaCl **(A)** and KCl **(B)** concentrations. The range of NaCl concentrations for YPCBV-1 proliferation **(C)** and the percent adsorption at different NaCl concentrations **(D)**. Error bars represent the standard deviation of three replicates. **(C)** has only one repetition.

The phage YPCBV-1 survived well in higher concentrations of NaCl (10% to saturation) and KCl (25% to saturation) but could not survive in pure water, indicating a strong dependence on NaCl or KCl (Figures 2A,B). YPCBV-1 can survive in NaBr concentrations ranging from 5 to 10% (w/v), and CaCl_2_ concentrations ranging from 1 to 10% (w/v; Supplementary Figure S5). YPCBV-1 survives in different concentrations of YPCBV-1 but only produces viral progeny at 5–20% (w/v), with an optimal salinity of 10% (w/v; Figure 2C). In addition, the adsorption efficiency of YPCBV-1 increased and then decreased with increasing NaCl concentrations. YPCBV-1 showed maximum adsorption efficiency at 5 and 8% (w/v) NaCl with 99.0 and 98.5% adsorption efficiency at 15 min, respectively. Ninety-four point nine % (±3%) of adsorption efficiency was retained up to 10% NaCl and dropped to 7.1% ± 1% at 30% NaCl (Figure 2D). Interesting, YPCBV-1 stability decreased sharply in pure water but 77% adsorption efficiency was retained. Although the maximum adsorption efficiency was found at 5–8% (w/v) NaCl, YPCBV-1 produced more progeny at 10–20% (w/v) NaCl, indicating higher adsorption efficiency do not produce more viral progeny and salinity affects the post-adsorption process.

### Effect of pH, temperature, organic solvent, detergents, and proteolytic degradation on YPCBV-1

**Figure 3:**
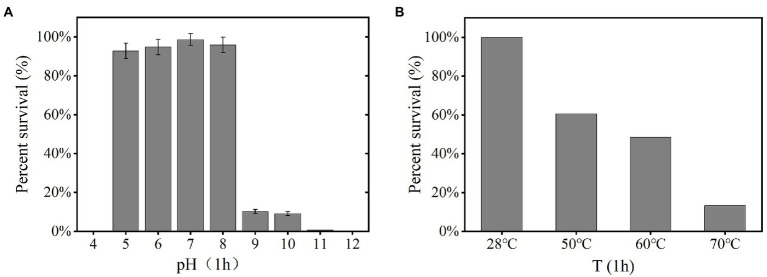
Tolerance of phage YPCBV-1 to pH (1 h; **A)**, temperature (1 h; **B)**. Error bars represent the standard deviation of three replicates and **(B)** has only one repetition.

The YPCBV-1 was stable at range of pH 5–10. The percent survival at pH 9 and pH 10 is much lower than at pH 5–8 and the optimal survival pH was 7, with a percent survival of 98.7% (Figure 3A). The percent survival of YPCBV-1 decreased with the increase of temperature, and the percent survival was 60.0% at 50°C for 1 h (Figure 3B).

YPCBV-1 was inactivated after 0.5 h treated with anhydrous ethanol. While, after 12 h incubation with chloroform, percent survival of phage YPCBV-1 was 40% ± 20%. In addition, the percent survival of YPCBV-1 was 73.1 ± 3 and 66.7% ± 8% after 1 h of treatment with proteinase K and SDS, respectively.

### Genome overview of YPCBV-1

**Figure 4:**
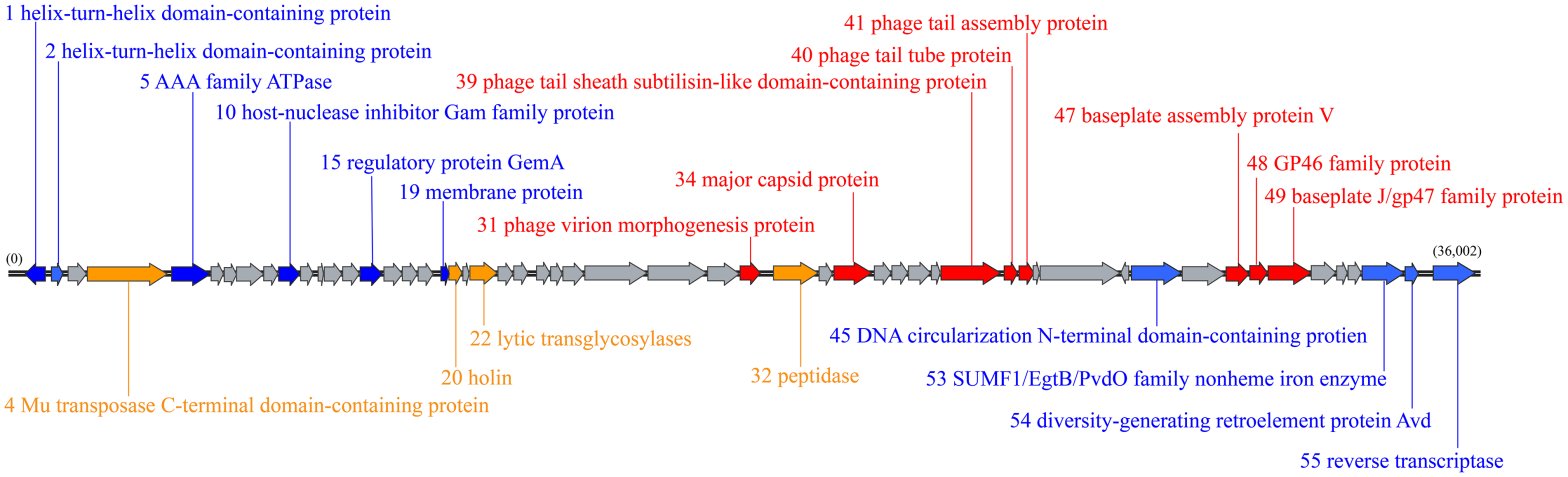
Genomic map of YPCBV-1. Gray represents predicted putative protein/DUF family protein genes; orange represents predicted lytic/lysogenic genes; blue represents predicted regulatory genes; and red represents predicted structural genes.

Sequencing reads of YPCBV-1 assembly resulted in two contigs. These two contigs was assembled as one scaffold but no terminal repeats were found. Overall, we considered that scaffold represents nearly complete (draft) genome of the phage YPCBV-1. The size of the draft genome of YPCBV-1 was 36,002 bp with a C + G content 67.09%. In total 55 ORFs were predicted in the genome, and the length of all ORFs was 34,101 bp, accounting for 94.74% of the genome. ORF1 and ORF44 were found on the negative strand, while the remaining 53 ORFs were found on the positive strand. No tRNA or rRNA gene was found in YPCBV-1 genome. Among all the predicted ORFs, the predominant start codon was AUG (50 ORFs), but there were also incidences of alternative start codons, i.e., UUG (ORF55) and GUG (ORF10, ORF21, ORF42, and ORF46). The predicted ORFs were compared with the GenBank nr database by Blastx (Supplementary Table S1). Except for ORF12, which was not annotated with any information, 24 ORFs were annotated as putative proteins, 8 ORFs were annotated as domain of unknown function (DUF) family proteins (Punta et al., 2011), and 22 ORFs were annotated with a clear function (Figure 4). Consistent with the results of many previous haloviruses, YPCBV-1 also has many genes with unknown functions. These proteins were compared with the Conserved Domains Database (CDD; Marchler-Bauer et al., 2002) to understand the conserved structural domains and their possible functions. Based on the predicted protein functions, the YPCBV-1 genome can be roughly classified into four major categories: structural, lytic/lysogenic, regulatory, and other genes.

### Structure and assembly of YPCBV-1

Among the structural genes, ORF34 is predicted to encode the major capsid protein, which is the main component of the phage capsid (Becker et al., 1997). In addition to the major capsid protein, other proteins which are important to stabilize the capsid. For example, there is a 9-kDa protein, P30, in phage PRD deletion of P30 leads to incomplete particle assembly and the formation of empty phage-specific membrane vesicles (Rydman et al., 2001). There is a pfam04233 structural domain in ORF30 of YPCBV-1, which belonging to the Phage_Mu_F superfamily, a minor head protein, implies that the ORF30 may be involved in YPCBV-1 capsid formation and play a possible role in stabilizing the capsid.

The phage tail is a very complex structure that plays an important role in host recognition, attachment, and cell membrane penetration (Leiman and Shneider, 2012). Among the 13 ORFs from 39 to 51, ORF42, 43, 44, 46, and 51 are predicted to encode putative proteins; ORF50 is predicted to encode a DUF2313 family protein; and other ORFs are predicted to encode tail proteins.

ORF39 and ORF40 encode tail sheath proteins and tail tube protein (TTP), respectively, which are important components of the tail and play an important role in the injection of DNA (Aksyuk et al., 2009; Zoued et al., 2016). In addition, the successful assembly of the tail requires the presence of a tail assembly chaperone (TAC; encoded by ORF41). Tape measure protein (TMP) is rapidly hydrolyzed if TAC is not present in phage P2 (Pell et al., 2013). TMP determines the length of the tail tube (Arnaud et al., 2017), and TAC is present between the genes that encode TTP and TMP (Maxwell and Davidson, 2013). ORF41 encodes TAC and ORF40 encodes TTP in YPCBV-1. The putative protein encoded by ORF42 have the possible function of TMP.

The baseplate is a key component of the tail that mediates host binding and genome injection (Büttner et al., 2016). ORF47, 48, and 49 in YPCBV-1 encode phage baseplate assembly protein V, phage GP46 family protein, and baseplate J/gp47 family protein, respectively. The protein encoded by ORF 48 has a pfam07409 structural domain, a GP46 protein (baseplate protein) similar to that of *Escherichia coli* phage Mu (Büttner et al., 2016). In addition, ORF43 encodes a putative protein with a COG5280 structural domain that belongs to the YQBO superfamily, a small-tailed protein associated with phage. ORF45 encodes a DNA cyclized N-terminal structural domain protein, which is a protein necessary for tail assembly as well as responsible for DNA injection.

In addition to the correct assembly of the phage prohead, packaging of the DNA is essential. ORF27 and ORF28 are predicted to encode the DUF3486 family protein, a terminase similar to *E.coli* phage Mu (Yuan et al., 2014). Meanwhile, ORF29 is predicted to encode the DUF935 family protein (Petrovski et al., 2011), which points to the portal protein in the CDD database. The portal protein plays an important role in the packaging process of DNA by transferring DNA into the prohead and is a central part of the DNA packaging motor (Isidro et al., 2004), as well as a key point between the head and tail proteins (Bazinet and King, 1985). In summary, ORF27, ORF28, and ORF29 may be involved in the packaging of DNA. Stable attachment of the tail to the head is mediated by the protein putatively encoded by ORF31.

### Lysis and lysogenesis of YPCBV-1

The lytic transglycosylase putatively encoded by ORF22 allow the genome to cross the cell wall without excessive harm to the host by locally expanding the gap in the peptidoglycan network (Lehnherr et al., 1998; Koraimann, 2003). YPCBV-1 releasing progeny may rely mainly on peptidases (Endolysin) putatively encoded by ORF32 (Pei and Grishin, 2005). In addition, endolysins have no signal sequence and require the presence of Holin to lyse the host cell wall (Wang et al., 2000, 2003; Fischetti, 2005; Catalão et al., 2013). In YPCBV-1, holin is putatively encoded by ORF20.

In phage Mu, the key to DNA integration in the host genome is the Mu transposase (MuA), which has 663 amino acids (Baker et al., 1993; Au et al., 2006). The protein putatively encoded by YPCBV-1 ORF4 (671 amino acids) is a Mu transposase C-terminal structural domain protein. In addition, DNA translocation of phage Mu is dependent on MuA, while MuB was found to play a role in regulating MuA activity as well as translocation as an ATP-dependent non-specific DNA-binding protein. This study also demonstrated that MuB is an AAA+ ATPase (Mizuno et al., 2013). It is speculated that the AAA family ATPase putatively encoded by ORF5 have MuB-like functions. To summarize, YPCBV-1 not only lyses the host to complete survival but also may have a lysogenic lifestyle.

### YPCBV-1-host interaction

ORF1 and ORF2 in the YPCBV-1 genome are predicted to encode helix-turn-helix (HTH) structural domain protein. HTH proteins have a short DNA-binding region that functions in many biological transcription processes (Rosinski and Atchley, 1999). The membrane protein putatively encoded by ORF19 then be involved in DNA replication (Meijer et al., 2010). ORF10 is predicted to encode a host nuclease inhibitor Gam family protein that keeps the YPCBV-1 genome from being lysed by inhibiting host nuclease activity. This is probably a protective mechanism arising from the long-term interaction between the phage and the host. ORF15 is predicted to encode the regulatory protein GemA, which is similar to the product encoded by GemA in the Gem operon in phage Mu, regulates the expression of a variety of host genes, including cell division and DNA replication as well as reducing host DNA gyrase activity and DNA relaxation (Ghelardini et al., 1994). In addition, some putative proteins may have regulatory roles, such as ORF18 is predicted to encode putative protein that has a pfam08765 structural domain and belongs to the Mor superfamily, a sequence-specific DNA-binding protein that mediates transcriptional activation by interacting with the C-terminal structural domains of the α and σ subunits of RNA polymerases.

We annotated to some specific proteins in the YPCBV-1 genome. Non-heme iron enzymes can catalyze a series of important metabolic transformations in various organisms (Lange and Que, 1998; Neidig and Solomon, 2005). It was putatively encoded by ORF53. In addition, two genes that can assemble diversity-generating retrotransposons (DGRs) are present in the YPCBV-1 genome, named ORF54 and ORF55, which are predicted to encode accessory variability determinant (bAvd) protein and reverse transcriptase (bRT), respectively. They can accelerate ligand–receptor interactions (Alayyoubi et al., 2013; Guo et al., 2014).

### YPCBV-1 Is a novel Mu-like phage

**Figure 5:**
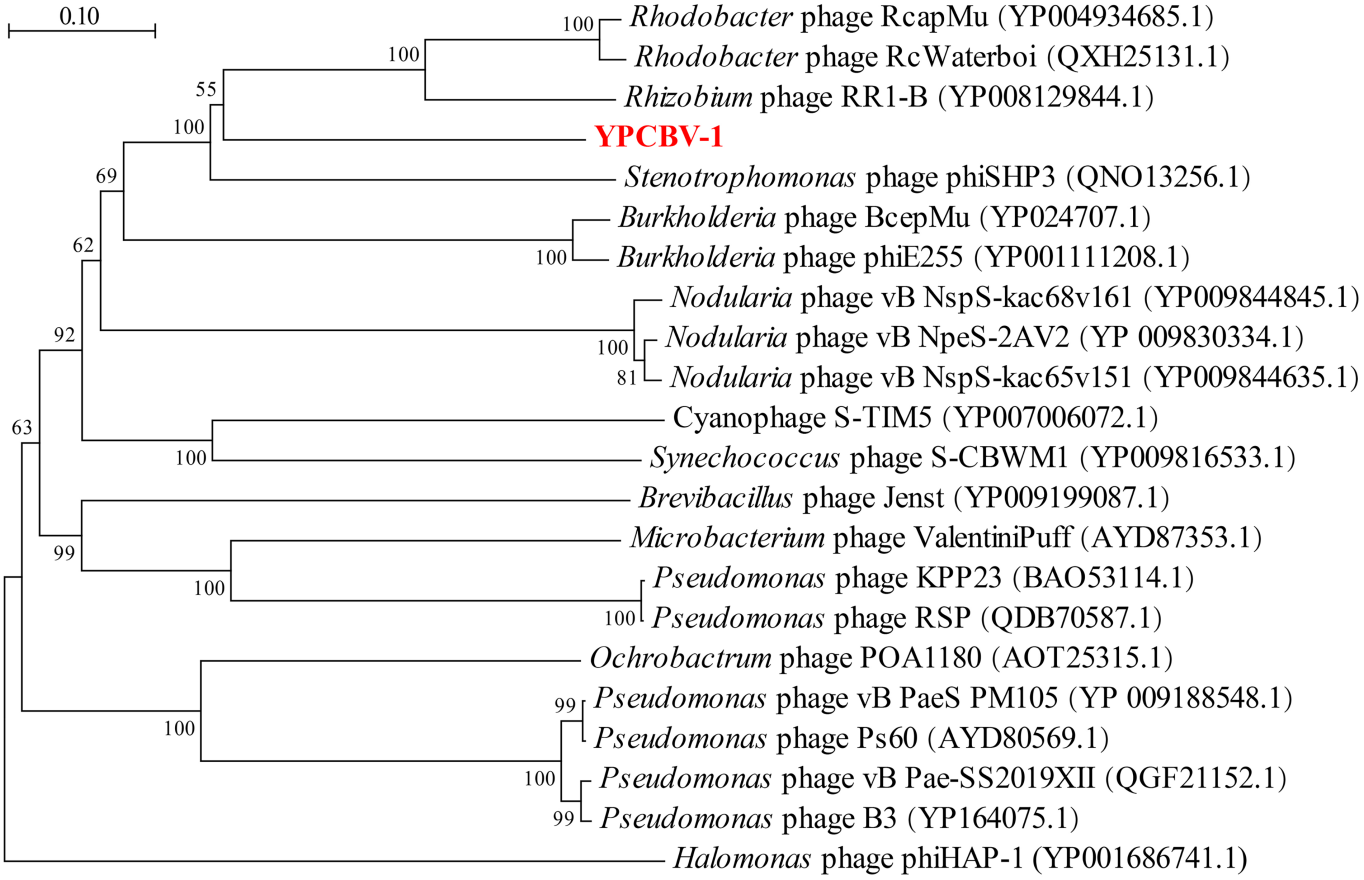
Major capsid protein phylogenetic tree. Phylogenetic trees were constructed using the neighbor-joining method by Mega 7.0. All parameters are default except the Bootstrap value is 1,000, the p-distance model is used to calculate the distance, and the Gap/Missing Data Treatment cutoff of 50%. Phage phiHAP-1 was selected as outgroup.

**Figure 6:**
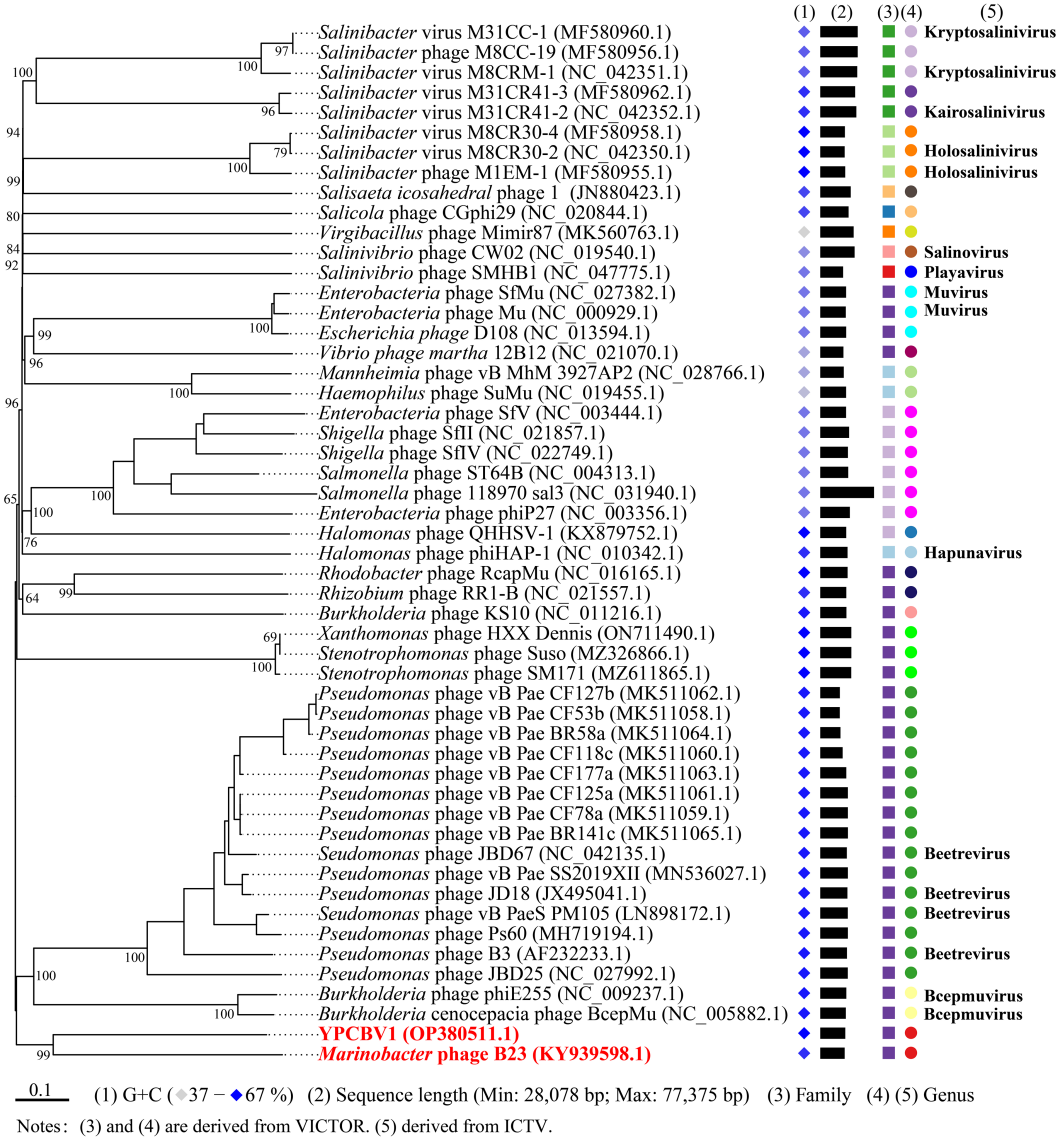
Phylogenetic analysis of the genome sequence of YPCBV-1 using VICTOR. The classification of family and genu is derived from VICTOR’s evaluation results. Squares represent families, circles represent genus, and different colors indicate different family/genus. Genus names were derived from ICTV (July 2021). Viruses with no genus name indicate that ICTV is not currently publishing their classification status.

**Figure 7:**
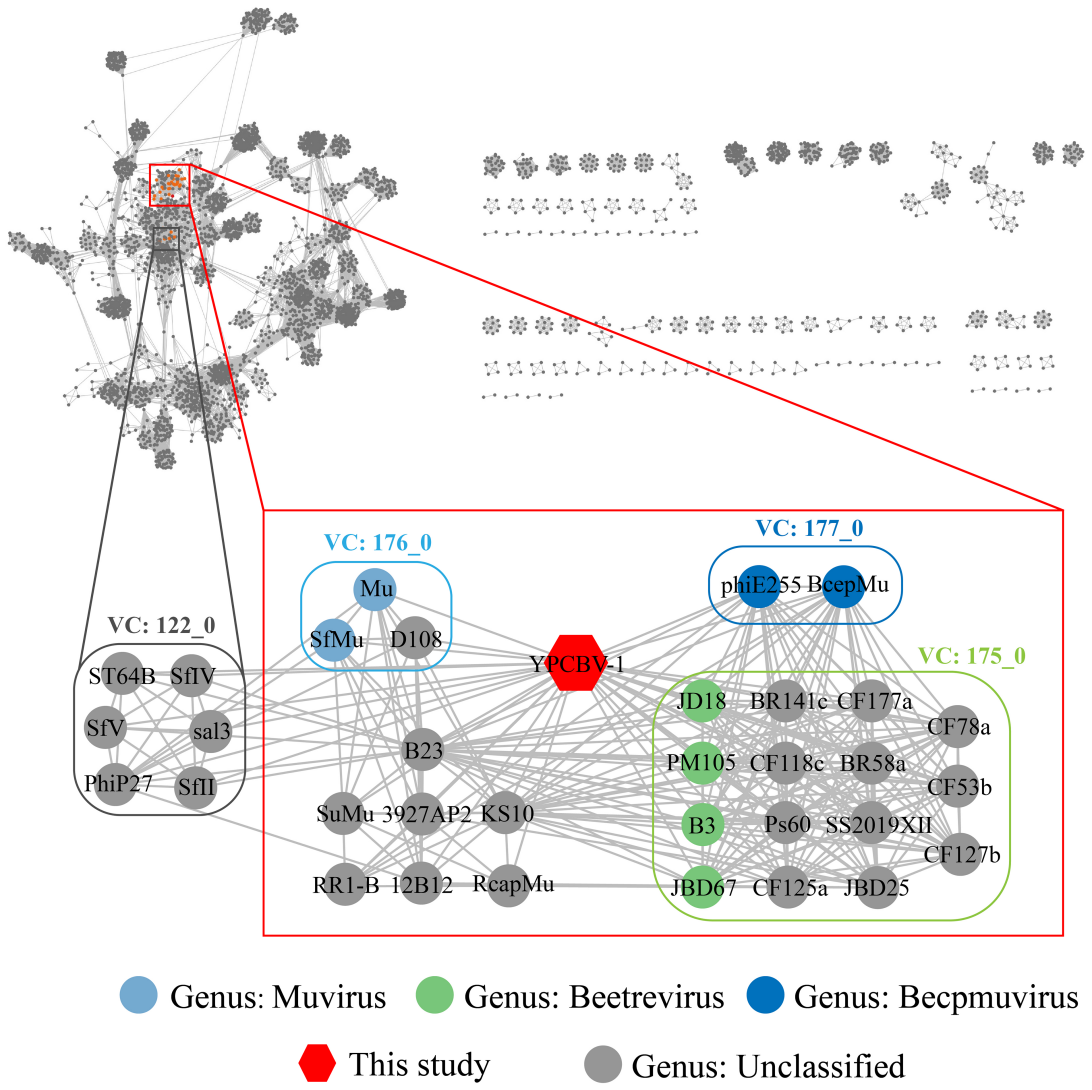
Genomic network diagram. Genomic network analysis was performed by vConTACT2, ClusterONE, and Cytoscape. The reference database is Prokaryotic Viral RefSeq211-Merged (Last updated in June 2022) and the top 20 similarity sequences in Genbank were added. Duplicate and unassociated sequences are removed. Edges indicate the presence of correlation between viral sequences, and virus clusters are shown as differently colored boxes. The classification status of viruses is derived from the ICTV (July 2021).

MCP, terminase, etc. are commonly used for virus classification (Rodela et al., 2019; Kwon et al., 2020; Olonade et al., 2021). Therefore, a phylogenetic tree was constructed based on the MCP of YPCBV-1 and 20 best hit viruses in BLASTp. The results shown that YPCBV-1 grouped with Mu-like phages but presents a separate branch and is far from the other sequences (Figure 5). In the whole genome phylogenetic analysis based on 20 best hit viruses in BLAST and 15 halobacterial viral genomes, YPCBV-1 and *Marinobacter* phage B23 (KY939598) clustered together and form a large group with halobacterial viruses (Figure 6). *Marinobacter* phage B23 was isolated from seawater and has a 35,132 bp genome with the G + C content of 59.8% (Zhu et al., 2018). The relatedness between YPCBV-1 and other viruses was further confirmed by genomic network analysis using vConTACT2. The results shown that 33 viruses have direct association with YPCBV-1 and most of them are Mu-like phages, such as B3, Mu, BcepMu, phiE255, RcapMu, etc. Although YPCBV-1 and 33 viruses were related within the network, YPCBV-1 was not classified into any of the virus clusters (Figure 7; Supplementary Table S2). In addition, nucleotide-based intergenomic similarities were calculated by VIRIDIC and showed the highest value of only 13.4% between YPCBV-1 and *Marinobacter* phage B23. Except B23, the average similarities between YPCBV-1 and VC175_0 (Beetrevirus) was the higher (average 4.63%; Supplementary Table S3). In conclusion, YPCBV-1 should be classified as a novel Mu-like phage under *Caudoviricetes.*

## Discussion

Salinity is the most important abiotic factor affecting viruses and their hosts in hypersaline environments (Kukkaro and Bamford, 2009; Junger et al., 2018). In this study, YPCBV-1 produced progeny within a NaCl concentration of 5–20% (w/v), and the optimal salinity was 10% (w/v), similar to the soil sample salinity (12.1%; Xiao et al., 2013). The burst size of YPCBV-1 at 5% (w/v) NaCl was lower than that of 8–20% (w/v) NaCl, but the highest percent adsorption (99%) was observed at 5% (w/v) NaCl, indicating that the high percent adsorption was not accompanied by a high burst size. Future studies should focus on the molecular mechanisms by which salinity affects virus infection of hosts.

Although YPCBV-1 produce progeny at NaCl concentrations of 5–20% (w/v), it can survive for longer times in different concentrations of NaCl or KCl ranging from 1% (w/v) to saturation. YPCBV-1 has a broader salinity adaptation compared to the host, which is similar to SCTP-1, SCTP-2, and QHHSV-1 (Kukkaro and Bamford, 2009; Fu et al., 2016).

These results suggested that low salinity is more suitable for host growth, while medium salinity is more favorable for halovirus YPCBV-1 to infect the hosts, and high salinity is favorable for halovirus YPCBV-1 survival. This characteristic may be the result of the adaptation of halovirus to the natural hypersaline environment, where the decrease in salinity due to precipitation promotes rapid host growth, and when drought occurs, salinity increases and halovirus infects the host and produce progeny, which enables them to survive in the environment for a longer time. Although YPCBV-1 can survive in different concentrations of NaCl and KCl, it can only survive in 5–10% (w/v) NaBr and 1%–10% (w/v) CaCl_2_. The mechanism of the different responses of YPCBV-1 to different ions is also well worth further investigation.

YPCBV-1 was found to form plaques in F3 lawn, indicating the existence of a lytic lifestyle. However, lysogenicity-related genes were also found in the genome, suggesting that YPCBV-1 may have both lysogenic and lytic lifestyles, and the existence of this feature may greatly improve the chances of survival of YPCBV-1. Salinity may be the switch that regulates the life cycles of halovirus (Atanasova et al., 2016). For example, haloarchaeal virus SNJ1 survives in the host as a lysogen at a salinity of 18% (w/v) and lysis at a high salinity of 25%–30% (w/v; Mei et al., 2015).

YPCBV-1 form one cluster with phage B23 in the whole genome phylogenetic analysis with 13.36% similarity (calculated by VIRIDIC). Marinobacter phage B23 was isolated from Bohai Sea, China, with a genome of 35,112 bp and GC% content of 59.8% (Zhu et al., 2018). YPCBV-1 has a larger genome (36,002 bp) and higher GC% content (67.9%) than phage B23. However, genomic network analysis did not classify them into one VC. In addition, MCP phylogenetic analysis and genomic network analysis revealed that YPCBV-1 is associated with many Mu-like phages (B3, Mu, SfMu, BcepMu, phiE255, etc.). The above results shown that YPCBV-1 may be a novel Mu-like phage close to Marinobacter phage B23 under Caudoviricetes. It is noteworthy that phages related to YPCBV-1 such as B3 (siphovirus), Mu (myovirus), and Becpmu (myovirus) have different morphologies. Hulo et al. (2015) proposed classifying these phages as Saltoviridae, with tail morphology as a subfamily. In the whole genome phylogenetic analysis of YPCBV-1, phages such as B3, Mu, Becpmu, and YPCBV-1 were evaluated as a family. And these phages were also found to form a cluster (probably at the family level) in the genomic network analysis. These results suggest that the classification of phages into a family by tail morphology may not be appropriate. A more comprehensive system is needed for virus classification.

A recent study analyzing a dataset of more than 30,000 DGRs in public metagenomics established six major DGR lineages, three of which are predominantly encoded by phages and appear to be used to diversify host attachment proteins, demonstrating that DGRs are broadly active and responsible for more than 10% of amino acid changes in some organisms (Roux et al., 2021). These results suggested that the presence of DGR in YPCBV-1 will be highly resilient to unpredictable hosts, environments, and so on.

In addition, we identified genes putatively encoding SUMF1/EgtB/PvdO family non-heme iron enzymes in the YPCBV-1 genome. Ergothioneine synthase (EgtB) is a unique non-heme mononuclear iron enzyme that is a key step in ergothioneine synthesis (Goncharenko and Seebeck, 2016; Tian et al., 2018). Ergothioneine is a natural antioxidant with special physicochemical properties and a specific distribution that make it significantly more stable than common antioxidants and comparable to conventionally recognized antioxidants such as glutathione and ascorbic acid (Huang and Donk, 2015; Cumming et al., 2018; Halliwell et al., 2018). In summary, these results imply that YPCBV-1 infection of the host may enhance host antioxidant activity and strengthen host survival in hypersaline environments, which is beneficial not only for host growth but also for the survival of the halovirus itself. Of course, the pros and cons are interchangeable, and this property may also lead to the possibility that some harmful microorganisms may also acquire this ability through certain means such as horizontal gene transfer.

With the development of science and technology, many strain resources have been obtained, but most microorganisms in hypersaline environments cannot be cultured, and the majority of haloviruses and their hosts are still unknown (Atanasova et al., 2015a). Therefore, strengthening the isolation and characterization of haloviruses is one of the important objectives in the future, which will provide experimental materials for studying halovirus–host interactions and physiological and biochemical characteristics and contribute to the discovery of new genes and enzymes. As predators feeding mainly on prokaryotes in hypersaline environments (Pedrós-Alió et al., 2000), haloviruses have a very important role in the ecological functions. Therefore, it is also important to study the roles of haloviruses in regulating biomes, mediating gene transfer, and participating in the material cycle.

## Data availability statement

The data presented in the study are deposited in the GenBank repository, accession number OP380511.

## Author contributions

WX, XC, and ZL: concept design and designation of experiments. HY and CF: experiments performance. HY, CF, and KD: data analysis. HY and WX: manuscript writing. XC and WX: project administration. WX: funding acquisition. HY, CF, KD, ZL, XC, and WX: manuscript review. All authors contributed to the article and approved the submitted version.

## Funding

This work was supported by the National Natural Science Foundation of China (nos. 32071570 and 31200138), Yunnan Provincial Sciences and Technology Department (no. 2018IA100), and Major Science and Technology Projects of Yunnan Province (no. 202002AA100007).

## Conflict of interest

The authors declare that the research was conducted in the absence of any commercial or financial relationships that could be construed as a potential conflict of interest.

## Publisher’s note

All claims expressed in this article are solely those of the authors and do not necessarily represent those of their affiliated organizations, or those of the publisher, the editors and the reviewers. Any product that may be evaluated in this article, or claim that may be made by its manufacturer, is not guaranteed or endorsed by the publisher.

## Footnotes

^1^ http://www.ezbiocloud.net/identify

^2^ https://ggdc.dsmz.de/victor.php

^3^ https://github.com/tseemann/barrnap

^4^ snapgene.com
