# Supplementary material for: Characterization and genomic analysis of a novel halovirus infecting Chromohalobacter beijerinckii
Source: Front Microbiol. 2022 Dec 7;13:1041471. doi: 10.3389/fmicb.2022.1041471 (PMC9769972; doi:10.3389/fmicb.2022.1041471)
Supplement: Supplementary file 4 [file Data_Sheet_1.DOCX]

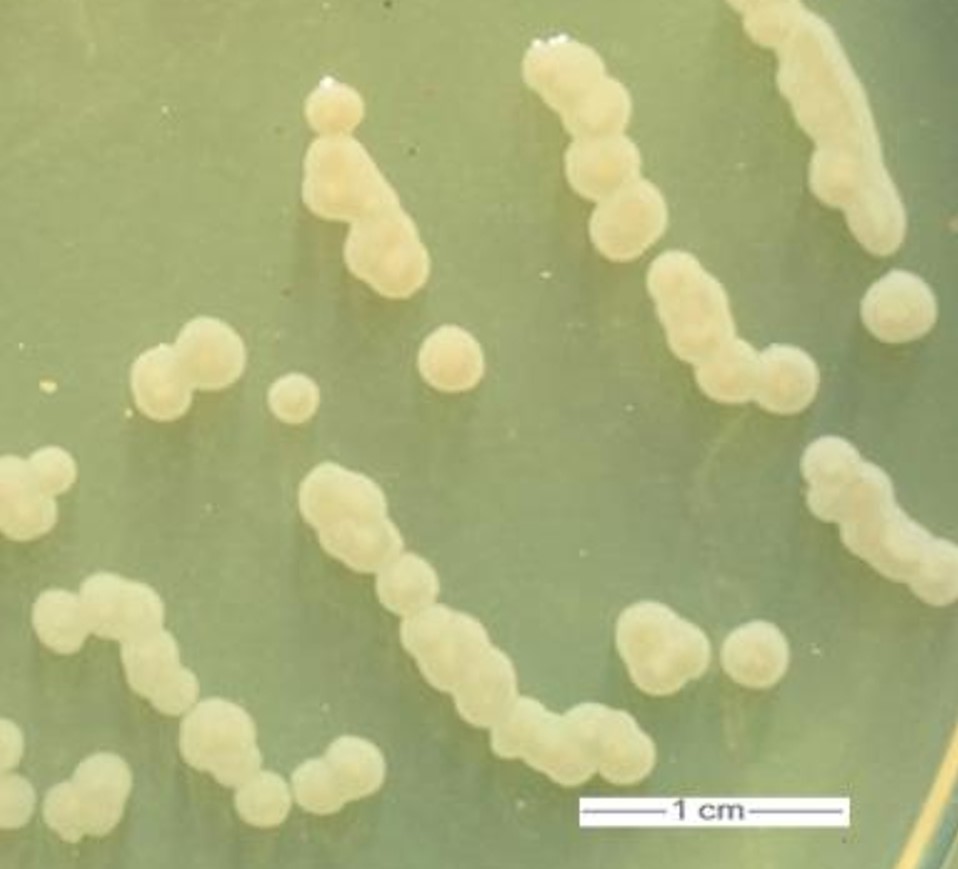


**Supplementary Figure 1** F3 colonies on plate.


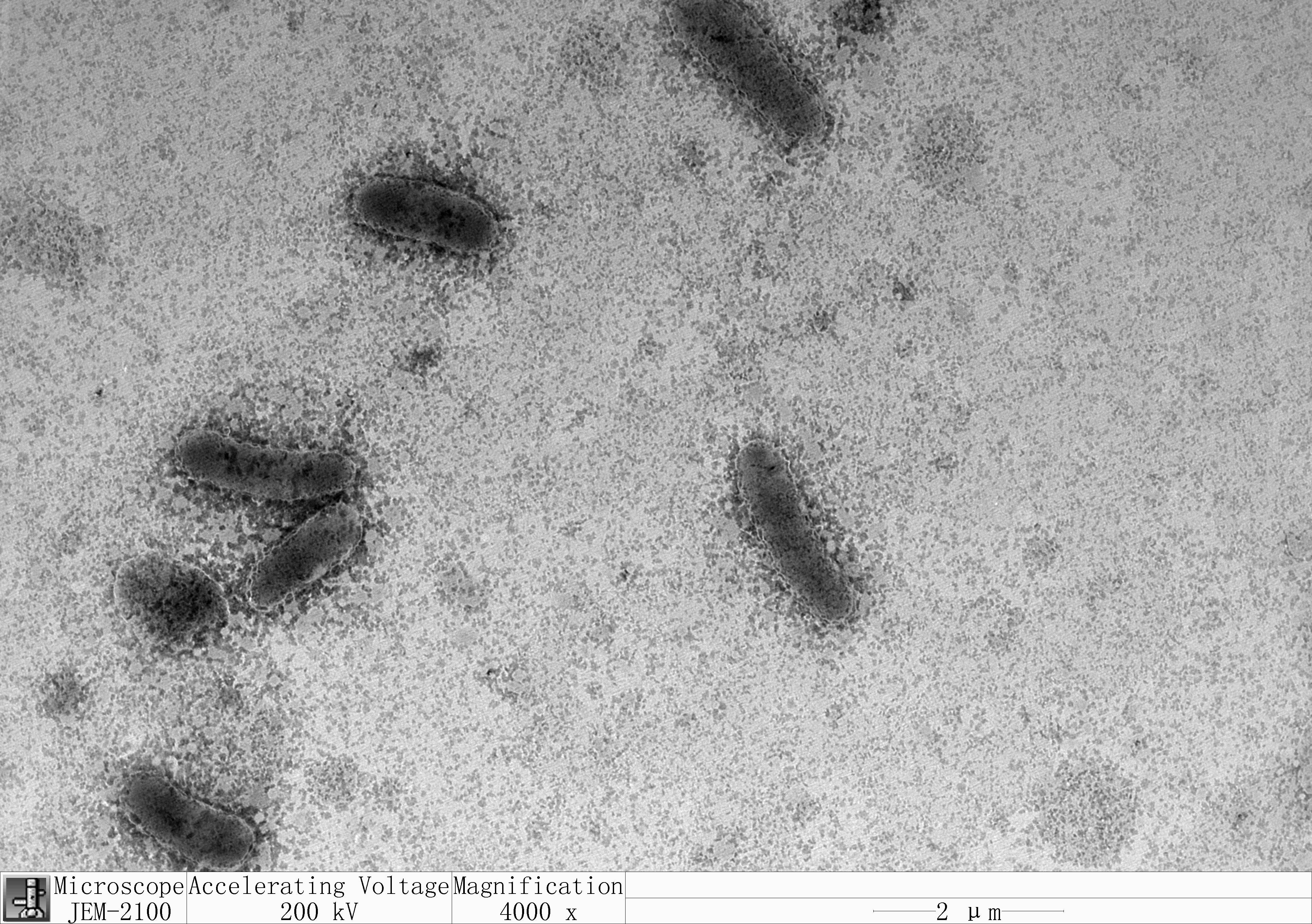


**Supplementary Figure 2** TEM micrographs of F3.


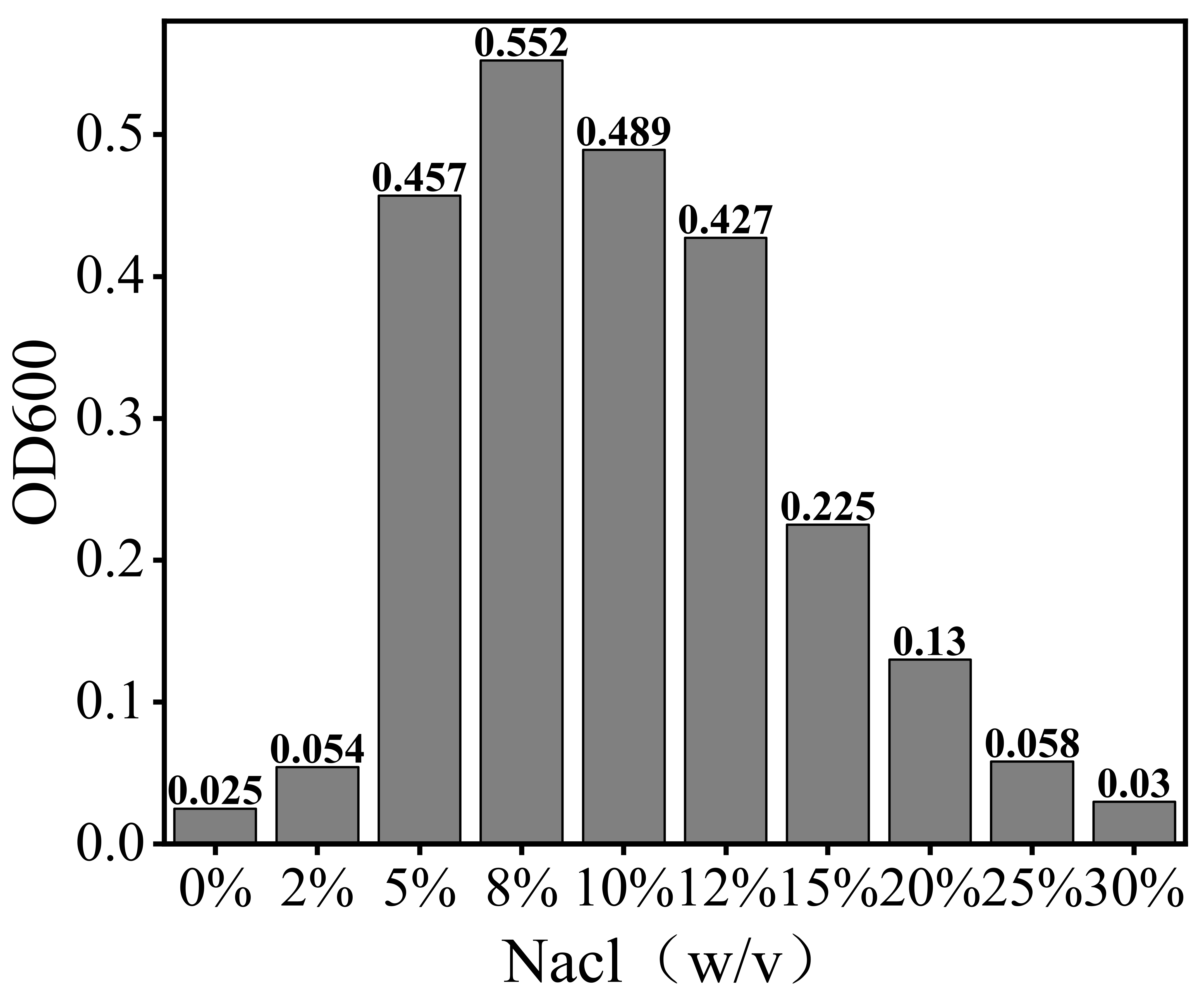


**Supplementary Figure 3** OD600 of host F3 at various salinity after 10h (one repetition).


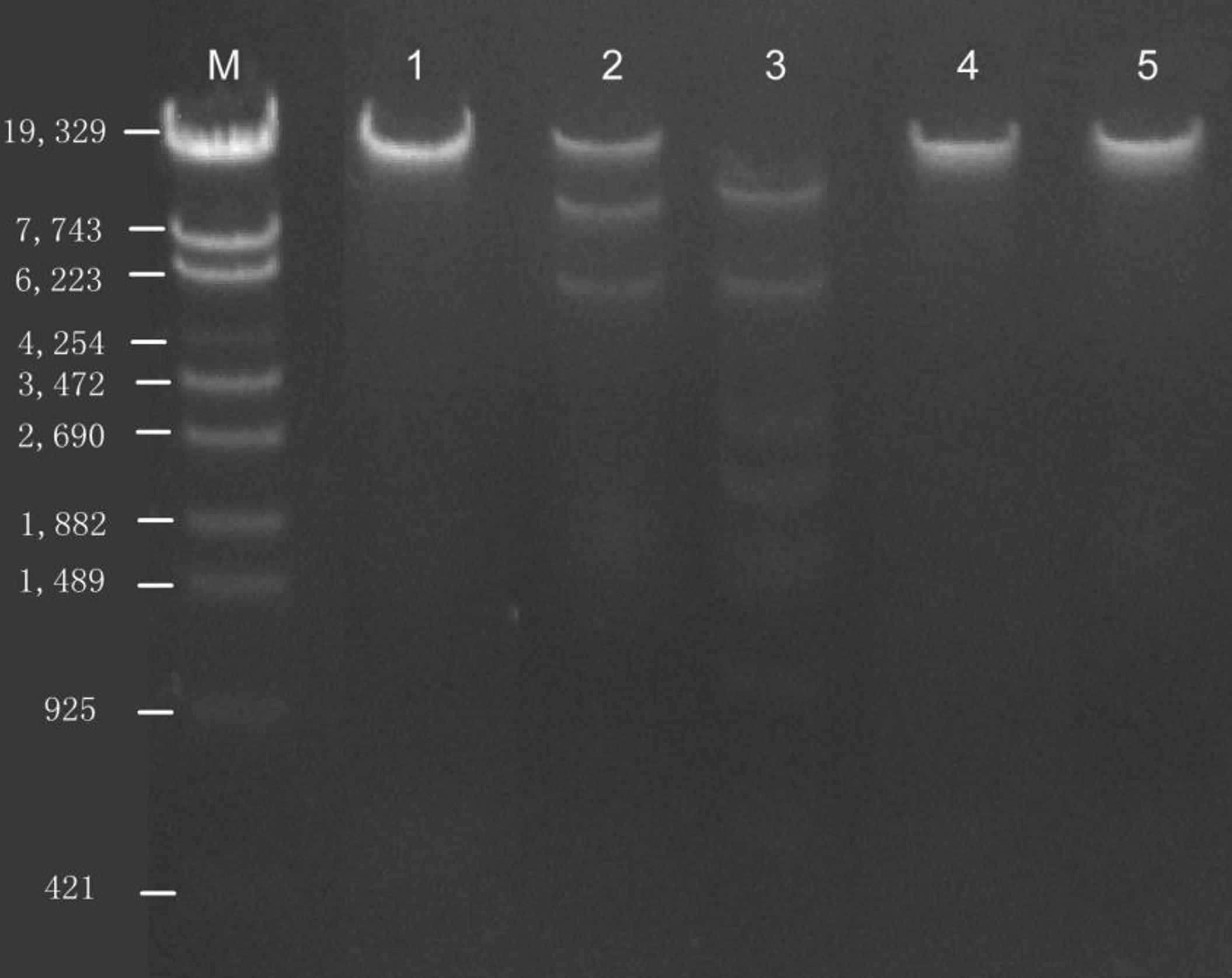


**Supplementary Figure 4** Restriction endonuclease analysis of YPCBV-1 DNA (M: Marker; lane 1, YPCBV-1 DNA; lane 2, YPCBV-1 DNA digested with *Eco*R I; lane 3, YPCBV-1 DNA digested with *Xho* I; lane 4, YPCBV-1 DNA digested with *Bam*H I; and lane 5, YPCBV-1 DNA digested with *Hind* III).


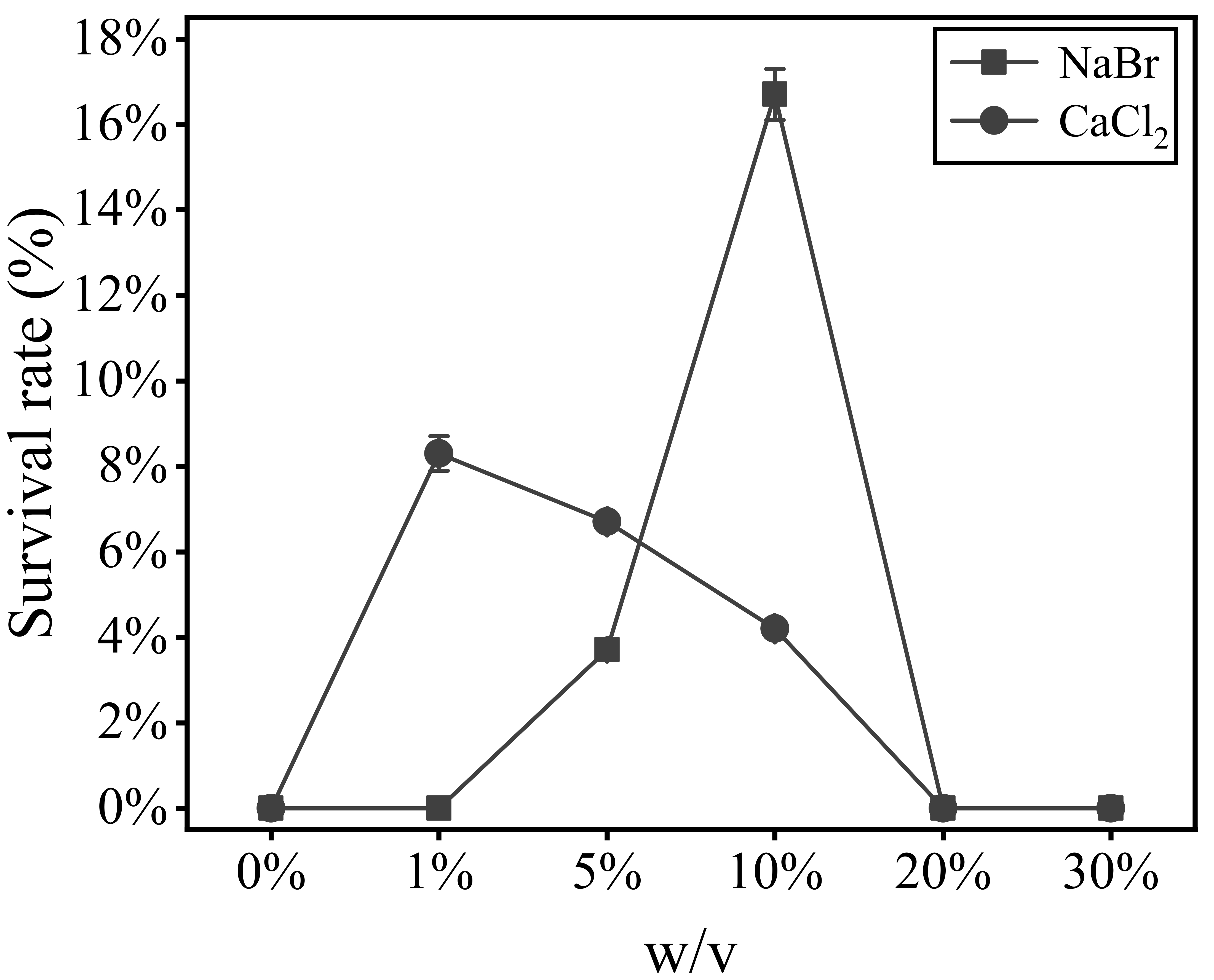


**Supplementary Figure 5** Percent survival of YPCBV-1 at various NaBr and CaCl_2_ concentrations (28d). Error bars represent the standard deviation of three replicates.
